# Competitive Antagonism of Xylazine on α7 Nicotinic Acetylcholine Receptors and Reversal by Curcuminoids

**DOI:** 10.1021/acschemneuro.4c00784

**Published:** 2024-12-25

**Authors:** Qiang Chen, Yan Xu, Pei Tang

**Affiliations:** † Department of Anesthesiology and Perioperative Medicine, University of Pittsburgh, Pittsburgh, Pennsylvania 15260, United States; ‡ Department of Structural Biology, University of Pittsburgh, Pittsburgh, Pennsylvania 15260, United States; § Department of Pharmacology and Chemical Biology, University of Pittsburgh, Pittsburgh, Pennsylvania 15260, United States; ∥ Department of Physics and Astronomy, University of Pittsburgh, Pittsburgh, Pennsylvania 15260, United States

**Keywords:** (1−6), α7 nAChRs, xylazine, curcuminoids, demethoxycurcumin, ivermectin, PNU120596

## Abstract

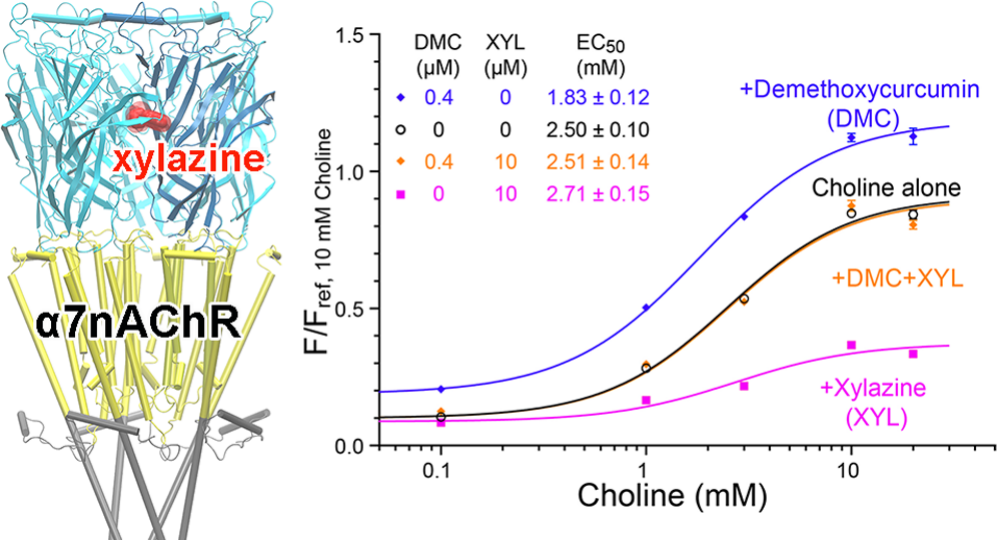

Co-use of xylazine with opioids is a major health threat in the United States. However, a critical knowledge gap exists in the understanding of xylazine-induced pharmacological and pathological impact. Xylazine is mostly known as an agonist of α2-adrenergic receptors (α2-ARs), but its deleterious effects on humans cannot be fully reversed by the α2-AR antagonists, suggesting the possibility that xylazine targets receptors other than α2-ARs. Here, we report the discovery of α7 nicotinic acetylcholine receptors (α7 nAChRs) as targets of xylazine. In *Xenopus* oocytes expressing α7 nAChRs, xylazine competitively antagonizes channel currents elicited by the agonist acetylcholine. In PC12 cells, xylazine suppresses choline-stimulated intracellular calcium ([Ca^2+^]_in_) transients that are mediated by endogenously expressed α7 nAChRs. Furthermore, we find that curcuminoids, ivermectin, and the α7-specific positive allosteric modulator PNU120596 can effectively offset the xylazine inhibition of α7 nAChRs. Considering the prominent role of α7 nAChRs in the cholinergic anti-inflammatory pathway and wide expression in the human body, our findings present a potential new strategy to reverse xylazine-caused damage using curcuminoids or repurposing ivermectin. This α7 nAChR-focused strategy may offer an immediate deployment that is likely effective in improving xylazine-related treatment outcomes.

## Introduction

The recreational use of xylazine-adulterated drugs, commonly fentanyl, has become a threat to human health in the United States.^1,2^ According to the CDC, drug poisoning deaths involving xylazine increased 1,238% from 2018 to 2021. However, no US Food and Drug Administration (FDA)-approved medications can effectively reverse xylazine’s damaging effects, such as central nervous system depression, agitated withdrawal, and skin ulcers and abscesses that lead to infections, rotting tissue, and even amputations.^1,3−6^ Although xylazine is known as an agonist of α2-adrenergic receptors (α2-ARs),^7^ none of the α2-AR antagonists provide full reversal of xylazine-induced deleterious effects.^1,3−6^ It was speculated previously that xylazine might also act on cholinergic and other receptors,^8^ but the supporting data are absent. The limited pharmacological understanding of xylazine and the lack of effective reversal medications have hampered the efficient clinical responses to this emerging threat. There is an urgent need to broaden our knowledge about the pharmacological and pathological impacts of xylazine. It is crucial to establish alternative therapeutic approaches targeting different biological pathways that can effectively counteract xylazine and improve treatment outcomes.

Among all potential targets of xylazine, α7 nicotinic acetylcholine receptors (α7 nAChRs) stand out for several reasons. First, α7 nAChRs are a major player in the cholinergic anti-inflammatory pathway.^9,10^ They are relevant to counter xylazine-caused damage, such as soft-tissue ulceration and inflammation.^11^ Agonists and positive allosteric modulators (PAMs) of α7 nAChRs are promising for the treatment of various inflammatory diseases.^12,13^Second, α7 nAChRs are expressed widely across the human body in both neural and non-neural cells, including skin cells.^9,12,14−18^ α7 nAChRs conduct higher Ca^2+^ current than other nAChR subtypes.^19^ They mediate both ionotropic and metabotropic signaling^13,20−22^ and are involved in a broad spectrum of actions in the central and peripheral nervous systems^14,23,24^Third, the promise of the cholinergic system in combating the opioid use disorder epidemic is well demonstrated in clinical and preclinical studies.^25,26^Finally, our results reported here provide compelling evidence supporting α7 nAChRs as a relevant xylazine target. Xylazine-induced changes in the function of α7 nAChRs can lead to observed harmful manifestations.

In addition to establishing α7 nAChRs as targets of xylazine, this study has also explored compounds that can offset xylazine effects. Natural products are well recognized for their roles in pharmacotherapy.^27^ Curcuminoids (including curcumin, demethoxycurcumin (DMC), and bisdemethoxycurcumin) are a small class of naturally occurring compounds isolated from turmeric. They are regarded by the FDA as “generally recognized as safe” (GRAS).^28^ Clinical trials show good tolerability and safety profiles, even at high doses.^29,30^ In clinical and preclinical studies, curcuminoids demonstrate anti-inflammatory, antioxidant, antihypertensive, neuroprotective, and pro-cognitive effects.^31−37^ They have been used to manage medical conditions occurring in many illicit drug users, including inflammatory and pain conditions, metabolic syndrome, and anxiety and depressive disorders.^38−41^ A strong correlation between opioid withdrawal and inflammation in the brain and gut has been observed.^42,43^ Targeting inflammation may alleviate the negative experience of drug withdrawal and prevent dependence.^42,43^ Curcumin reverses nociception in mouse models of inflammatory pain by acting as a PAM of α7 nAChRs.^44^ With their proven safety and efficacy, as well as the new experimental data presented in the current study, curcuminoids are likely to offer a promising alternative to counter xylazine-caused damage.

## Results

### Xylazine Competitively Antagonizes α7 nAChRs

**Figure 1:**
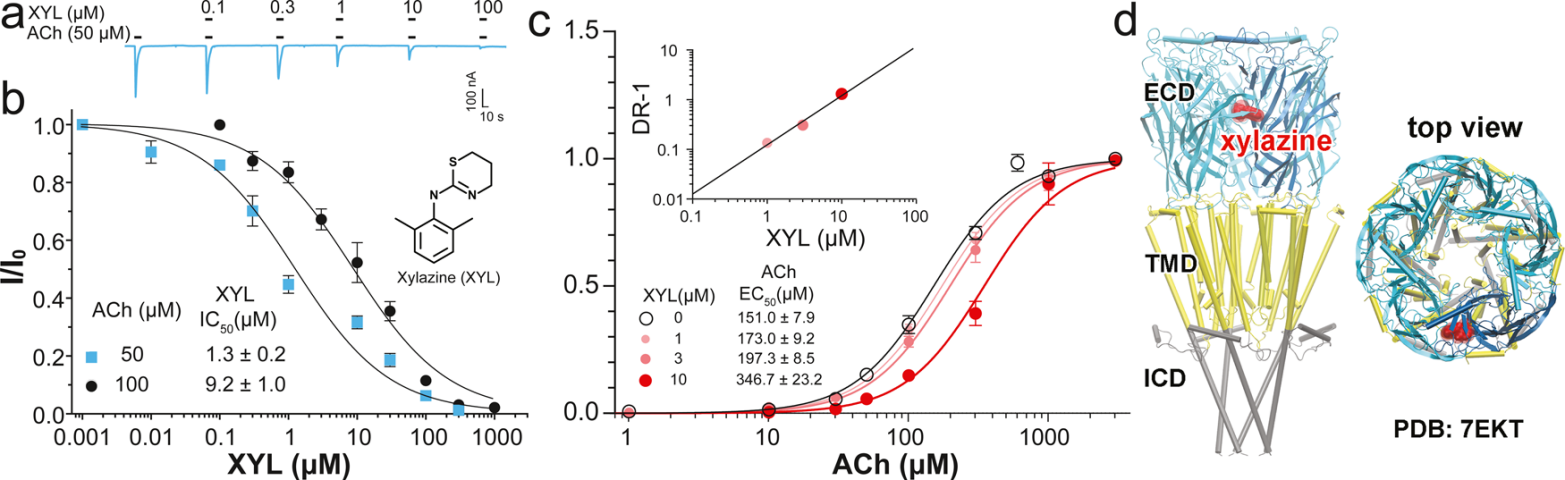
Xylazine is a competitive antagonist of α7 nAChR. (a) Representative traces showing xylazine (XYL) inhibition of currents elicited by acetylcholine (ACh) in *Xenopus* oocytes expressing α7 nAChR. (b) XYL concentration-dependent inhibition of α7 nAChR currents elicited by ACh. Inhibitory response is expressed as the fraction of current induced in the presence (*I*) of the indicated concentrations of XYL relative to that in the absence (*I*_0_) of XYL. The data fit to the Hill equation and resulted in XYL half-maximal inhibitory concentrations (IC_50_) as shown in the figure. (c) ACh concentration-dependent curves for α7 nAChR activation in the absence and presence of XYL, showing a right shift of EC_50_ with increased concentrations of XYL. These data were used to generate the inserted Schild plot, for which the Gaddum/Schild nonlinear regression fitting resulted in a Schild slope of 0.95 (*R*^2^ = 0.98), indicating XYL is a competitive antagonist. The fitting also shows a binding affinity of 8.4 μM for XYL onto α7 nAChR. All data in (c) are normalized to the maximum current of α7 nAChR activated with 3 mM ACh. All data in (b,c) are reported as the mean ± SEM from *n* ≥ 7 *Xenopus* oocytes from four donors. (d) Representative view of xylazine (red) binding to the orthosteric site in the extracellular domain (ECD) of α7 nAChR based on AutoDock.

In *Xenopus* oocytes expressing human α7 nAChRs, acetylcholine (ACh) activates α7 nAChRs and elicits the channel current (Figure 1a). In the same experimental setting, the ACh-induced current is completely inhibited by α-bungarotoxin (125 nM, an α7 nAChR-selective antagonist), confirming that the observed current results from α7 nAChR activation (Figure S1). Xylazine inhibits the ACh-current in a dose-dependent manner (Figure 1b). Fitting of the xylazine inhibition curves to the Hill equation yielded IC_50_ values of 1.3 and 9.2 μM at ACh concentrations of 50 and 100 μM, respectively. Note that the reported xylazine EC_50_ values on α2-ARs are in a range of ∼2 to ∼7 μM.^45,46^ We further measured xylazine’s effects on ACh’s potency and efficacy. A right shift of the ACh EC_50_ values was observed when xylazine concentrations were increased (Figure 1c). These data were used to generate a Schild plot^47^ (insert, Figure 1c), for which the Gaddum/Schild nonlinear regression fitting resulted in a Schild slope of 0.95 (*R*^2^ = 0.98), indicating that xylazine is a competitive antagonist of α7 nAChRs. Consistent with the experimental finding, our docking studies show xylazine’s occupancy to the orthosteric site in the extracellular domain of α7 nAChRs (Figure 1d), where xylazine can compete with agonists for binding and effectively reduce the potency of agonists.

### Curcuminoids Counteract Inhibitory Action of Xylazine

**Figure 2:**
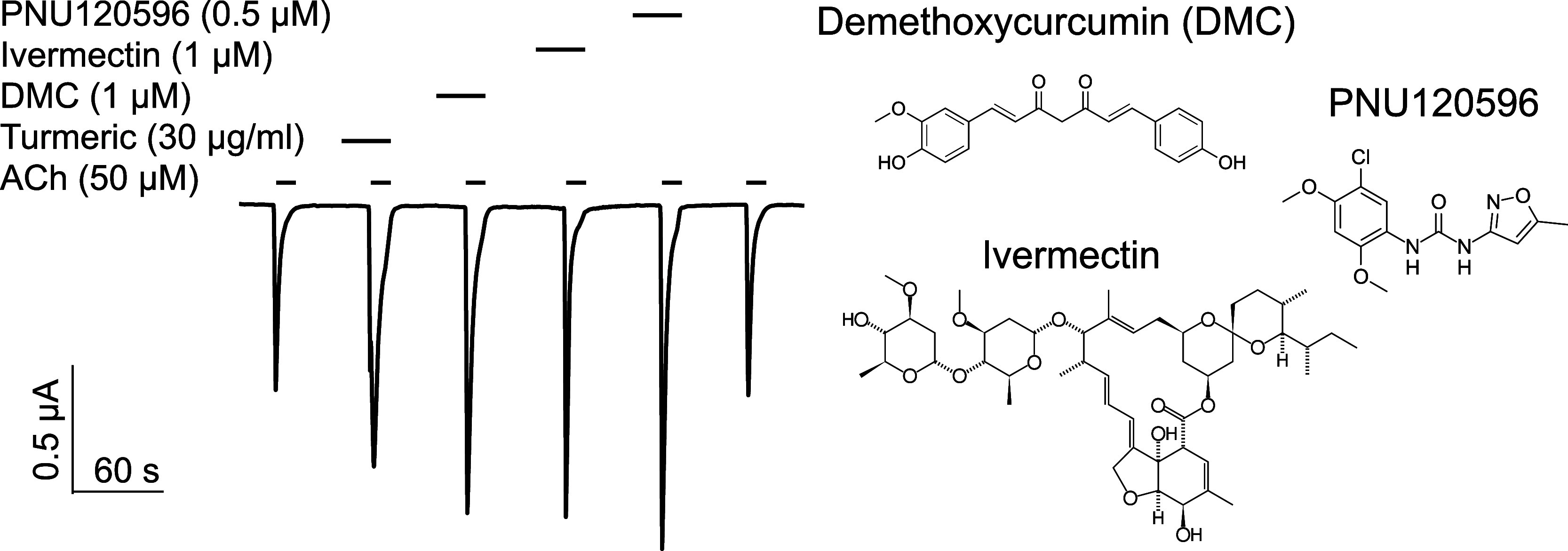
Representative trace showing α7 nAChR currents activated by acetylcholine (ACh, 50 μM) and potentiated by PAMs (turmeric, DMC, ivermectin, and PNU120596). All PAMs were applied 30 s earlier (preincubation) than the agonist ACh. The result was confirmed by different *Xenopus* oocytes (*n* = 5) expressing α7 nAChRs.

**Figure 3:**
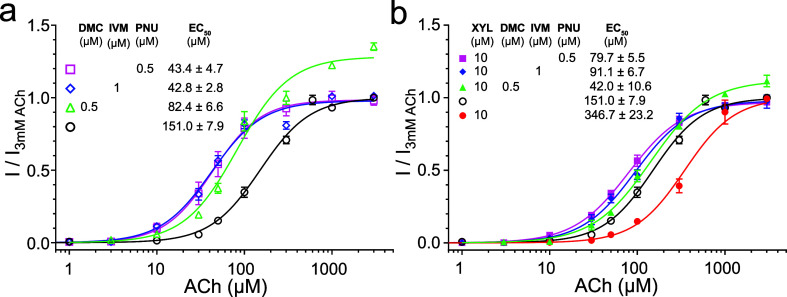
α7 nAChR PAMs can offset the inhibitory action of xylazine (XYL). (a) The α7 nAChR PAMs, including DMC (0.5 μM), ivermectin (IVM, 1 μM), and PNU-120596 (PNU, 0.5 μM), significantly increased the ACh potency (smaller EC_50_). DMC also increased the efficacy with a higher maximum plateau of *I*/*I*_3 mM ACh_ than that from the control. (b) The PAMs could effectively offset the inhibitory effects of XYL (10 μM, red). The α7 nAChR PAMs were used with a 30 s preincubation. Data in (a,b) are normalized to the α7 nAChR current activated by 3 mM ACh, fit to the Hill equation, and reported as the mean ± SEM from *n* ≥ 7 *Xenopus* oocytes and four different donors.

Turmeric is a natural product containing predominantly curcuminoids. Demethoxycurcumin (DMC) is a curcuminoid isolated from turmeric. Both are positive allosteric modulators (PAMs) of α7 nAChRs because they alone induce no detectable current but potentiate the current elicited by acetylcholine (Figure 2). We also tested the known PAMs of α7 nAChRs, ivermectin^48^ and PNU120596,^49^ and observed an expected potentiation effect (Figure 2). Further, we measured changes in the ACh potency and efficacy due to the presence of DMC (0.5 μM), ivermectin (IVM, 1 μM), or PNU120596 (PNU, 0.5 μM). These PAMs increased the potency of ACh, as indicated by the left shift of the α7 nAChR-activation curves and smaller EC_50_ values (Figure 3a). According to the extra sum-of-squares *F* test, these PAM-induced changes are significant [DMC: *F*(3,128) = 84.79, *p* < 0.0001; ivermectin: *F*(3,108) = 101.4, *p* < 0.0001; PNU120596: *F*(3,106) = 36.97, *p* < 0.0001]. DMC (0.5 μM) also increased the efficacy of ACh, as reflected by an elevated plateau of the maximum normalized current (Figure 3a). Finally, we tested whether these PAMs could offset the inhibitory effects of xylazine on α7 nAChRs. In the presence of 10 μM xylazine, all three PAMs can overcome the xylazine-introduced right shift and move the ACh dose–response curves closer to the control level (Figure 3b). The data also suggest that they can all offset xylazine effects even at a concentration lower than that currently used in Figure 3b. This finding is especially important for DMC and other curcuminoids that have a low bioavailability, which has been significantly improved due to recent advances in the formulation and delivery of curcuminoids. A new human pharmacokinetics study found a mean plasma curcumin concentration of ∼0.5 μM (*C*_max_ = 179.91 ng/mL),^50^ supporting the relevance of using DMC ≤ 0.5 μM to offset xylazine effects in our studies.

### Xylazine Suppresses Intracellular Calcium Transients Mediated by α7 nAChR

**Figure 4:**
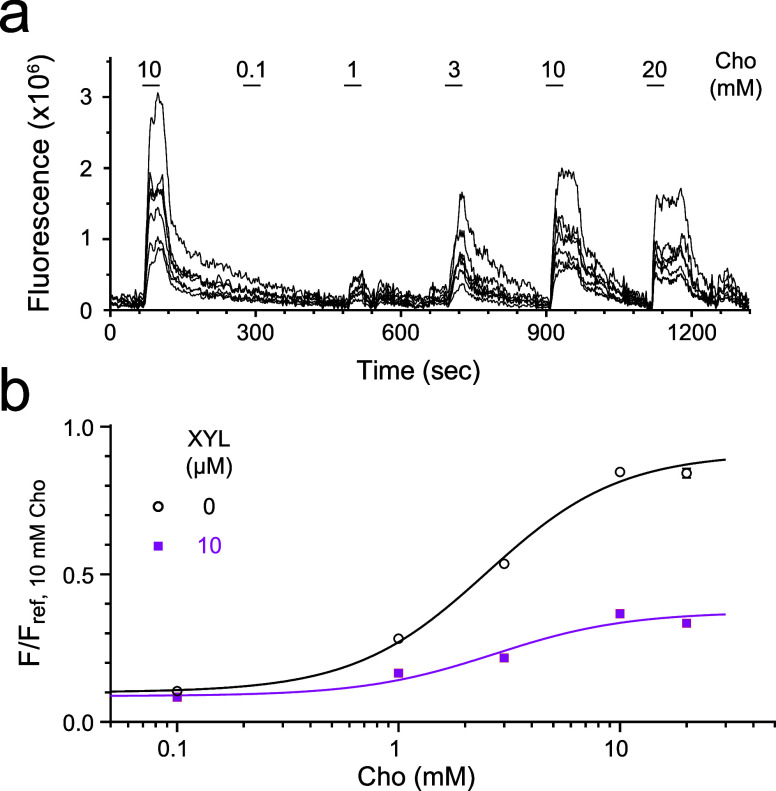
Xylazine inhibition of [Ca^2+^]_in_ transients elicited by choline (Cho) activation of endogenous α7 nAChRs in PC12 cells loaded with Calbryte 520 AM. (a) Representative traces of [Ca^2+^]_in_ fluorescent intensity stimulated by different concentrations of Cho. The fluorescence intensity resulting from the first 10 mM Cho application in each cell is defined as *F*_ref_ for normalizing fluorescence intensities under different Cho concentrations in (b). (b) [Ca^2+^]_in_ fluorescence responses as a function of Cho concentrations in the absence (black) and presence of xylazine (XYL, 10 μM, pink). The data fitting to the Hill equation resulted in Cho EC_50_ of 2.50 ± 0.10 or 2.71 ± 0.15 mM in the absence or presence of XYL, respectively. Data points are presented as mean ± SEM (*n* ≥ 130 cells). Most error bars are smaller than symbol sizes. Extra sum-of-squares *F* test [*F*(3,2396) = 1275, *p* < 0.0001] suggests a significant XYL inhibitory effect on Cho-stimulated [Ca^2+^]_in_ transients.

PC12 cells endogenously express α7 nAChRs but not α2-ARs.^51^ We performed live-cell fluorescent calcium imaging using PC12 cells loaded with Calbryte 520 AM (AAT Bioquest), a cell-permeable fluorescent indicator. Nifedipine (1 μM) was used to block potential interference from L-type Ca_v_ channels that are abundant in nondifferentiated PC12 cells.^52^ Upon activation of α7 nAChR by the α7-selective agonist choline (Cho), the intracellular calcium [Ca^2+^]_in_ transients increased (Figures 4 and S2) in a Cho-concentration-dependent manner with EC_50_ = 2.50 ± 0.10 mM and *E*_max_ = 0.91 ± 0.01, where *E*_max_ is the maximum plateau of *F*/*F*_ref_. The [Ca^2+^]_in_ transients stimulated by Cho were significantly inhibited by 10 μM xylazine (EC_50_ = 2.71 ± 0.15 mM and *E*_max_ = 0.37 ± 0.01) (Figures 4b and S2a) according to the extra sum-of-squares *F* test [*F*(3,2396) = 1275, *p* < 0.0001]. Similar results were also observed in PC12 cells loaded with Fura-2AM, where xylazine greatly suppressed [Ca^2+^]_in_ transients stimulated by ACh (Figure S3).

### Curcuminoids Potentiate Intracellular Calcium Transients and Counteract the Xylazine Inhibition

**Figure 5:**
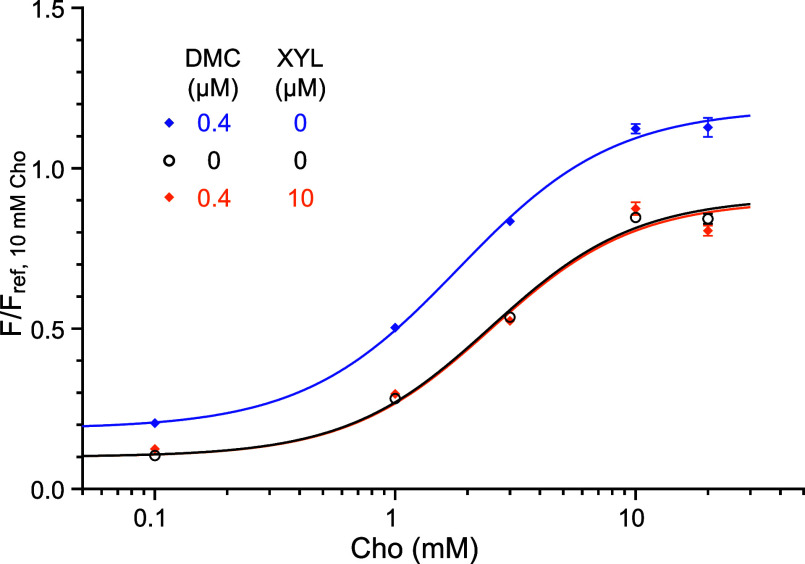
DMC potentiates [Ca^2+^]_in_ signals upon activation of α7 nAChRs by choline and counteracts the xylazine inhibitory effect. (a) Compared to the control (black), DMC (0.4 μM) significantly potentiated Cho-stimulated [Ca^2+^]_in_ transients (blue) with Cho EC_50_ = 1.83 ± 0.12 mM and *E*_max_ = 1.19 ± 0.03. The statistical significance of DMC potentiation is based on analyses of the extra sum-of-squares *F* test [*F*(3,1925) = 136.7, *p* < 0.0001]. (b) DMC (0.4 μM) reversed the xylazine inhibition (orange) and brought the [Ca^2+^]_in_ transients back to the control level, as indicated by the extra sum-of-squares *F* test [*F*(3,2339) = 0.2126, *p* = 0.8877]. The data were fit to the Hill equation. Data points are presented as mean ± SEM (*n* ≥ 130 cells).

Curcuminoids, including DMC, possess a native yellow color, whose autofluorescence can hinder the measurement accuracy of fluorescence intensity changes resulting from changes in the [Ca^2+^]_in_ transients. Curcuminoids’ yellow color falls out of the excitation and emission spectral range of Calbryte 520 AM (but not Fura-2). DMC at low concentrations shows a minimal background of fluorescence in PC12 cells loaded with Calbryte 520 AM (Figure S4). When α7 nAChRs were activated by Cho in the presence of 0.4 μM DMC, [Ca^2+^]_in_ transients were significantly potentiated (EC_50_ = 1.83 ± 0.12 mM and *E*_max_ = 1.19 ± 0.03) according to the extra sum-of-squares *F* test [*F*(3,1925) = 136.7, *p* < 0.0001] (Figures 5 and S2b). The DMC (0.4 μM) potentiation of α7 nAChR-mediated [Ca^2+^]_in_ transients counteracted the inhibitory effect of xylazine (10 μM) and brought [Ca^2+^]_in_ transients back to the control level with EC_50_ = 2.51 ± 0.14 mM and *E*_max_ = 0.9 ± 0.02 (Figures 5 and S2c), as indicated by the insignificant difference when compared with the control {the extra sum-of-squares *F* test [*F*(3,2339) = 0.2126, *p* = 0.8877]}.

### Xylazine Effects on Other Nicotinic Acetylcholine Receptors

**Figure 6:**
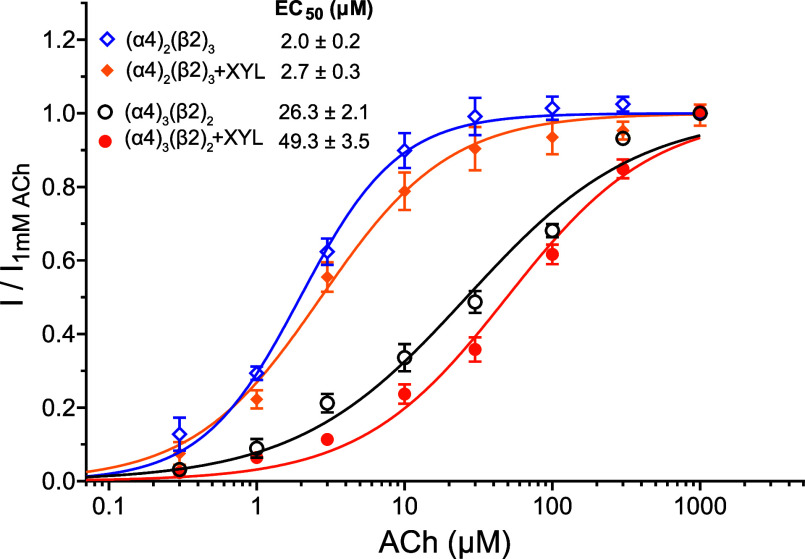
Xylazine effects on α4β2 nAChRs. The assemblies of (α4)_2_(β2)_3_ and (α4)_3_(β2)_2_ nAChRs show distinctly different responses to ACh in their ACh concentration-dependent curves. The presence of 10 μM xylazine induces a right shift of the concentration-dependent curves and elevation of ACh EC_50_ values in both assemblies. All data are normalized to the maximum current of (α4)_2_(β2)_3_ or (α4)_3_(β2)_2_ activated with 1000 μM ACh. All data are reported as the mean ± SEM from *n* ≥ 13 *Xenopus* oocytes from two donors.

α7 nAChRs are the primary cholinergic receptors responsible for mediating the anti-inflammatory cholinergic pathway that is closely related to countering xylazine-induced damage, including severe skin lesions and infection. Compared to α7 nAChRs, other subtypes of nAChRs play a less prominent role in the anti-inflammatory cholinergic pathway.^9,16,53−55^ Nevertheless, we performed electrophysiological measurements as shown in Figure 3 on the most abundant cerebral subtype α4β2 nAChRs, which exist in two different assemblies, (α4)_3_(β2)_2_ and (α4)_2_(β2)_3_, with the latter being more sensitive to agonists.^56^ Indeed, using *Xenopus* oocytes expressing human α4β2 nAChRs, we observed two distinct current responses to ACh stimulations, the higher-sensitivity (α4)_2_(β2)_3_ nAChRs (EC_50_ = 2.0 ± 0.2 μM) and the lower-sensitivity (α4)_3_(β2)_2_ (EC_50_ = 26.3 ± 2.1 μM) (Figure 6). Xylazine (10 μM) inhibits current responses to the agonist ACh and results in EC_50_ = 2.7 ± 0.3 and 49.3 ± 3.5 μM for (α4)_2_(β2)_3_ and (α4)_3_(β2)_2_, respectively. According to the extra sum-of-squares *F* test, the xylazine-induced changes are significant in both groups of assemblies [(α4)_2_(β2)_3_, *F*(2,177) = 4.8, *p* < 0.01; (α4)_3_(β2)_2_, *F*(2,162) = 17.6, *p* < 0.0001]. Comparing the two α4β2 assemblies, α7 experiences a stronger xylazine (10 μM) effect as reflected in an increase of ACh EC_50_ by ∼2.3 folds. In contrast, the same concentration of xylazine changes ACh EC_50_ values only by ∼1.4- and ∼1.9-fold on (α4)_2_(β2)_3_ and (α4)_3_(β2)_2_, respectively.

These experimental observations are supported by our xylazine docking studies. As in α7 nAChRs (Figure 1d), xylazine binds to the orthosteric ligand site in the extracellular domains of (α4)_3_(β2)_2_ and (α4)_2_(β2)_3_ (Figure S5a). Although residues near the xylazine binding site are largely similar among nAChR subtypes, the nonidentical residues are probably sufficient to alter xylazine effects on individual receptors (Figure S5b). A lower xylazine docking energy in α7 nAChRs than in (α4)_3_(β2)_2_ and (α4)_2_(β2)_3_ is consistent with the stronger negative impact of xylazine (10 μM) on ACh activation of α7 nAChRs (Figure 1c) than that of α4β2 nAChRs (Figure 6). We also performed xylazine docking onto α3β4 nAChRs that predicted the likelihood of xylazine action on α3β4 nAChRs (Figure S5). This prediction needs to be confirmed by future experiments.

Although DMC is an effective PAM of α7 nAChRs, it does not exhibit strong modulation of (α4)_3_(β2)_2_ and (α4)_2_(β2)_3_ nAChRs (Figure S6). This is also true for another curcuminoid, curcumin, which positively modulates α7 nAChRs but shows no effect on α4β2, α4β4, α3β2, and α3β4 nAChRs.^44^ These findings come as no surprise because hydrophobic PAMs (e.g., curcuminoids) bind to the transmembrane domains of the receptors, where binding pockets tend to have fewer conserved residues across different receptor subtypes compared with the orthosteric ligand binding site in the extracellular domain. Exact binding sites for DMC and other curcuminoids in α7 nAChRs are yet to be determined, but the binding sites revealed for ivermectin^57^ and PNU120596^58^ in the transmembrane domain of α7 nAChRs offer molecular insights into why these PAMs positively modulate the function of α7 nAChRs but do not impact functions of (α4)_3_(β2)_2_ and (α4)_2_(β2)_3_ nAChRs (Figure S7).

## Discussion

The current poor understanding of the pharmacological and pathological impact of xylazine on humans hampers the ability to treat patients with complicated use-dependence syndromes.^59^ Our study reveals that xylazine is a strong competitive inhibitor of α7 nAChRs. This discovery is significant in at least two respects. First, the recreational use of xylazine-adulterated drugs has induced symptoms beyond those caused by opioid misuse that require new treatment strategies. α2-ARs are known xylazine receptors, but not a single antagonist of α2-ARs can fully reverse xylazine-caused damage to human health, including skin ulcers. It is noteworthy that the EC_50_ values of xylazine on α2-ARs are in a range of ∼2 to ∼7 μM,^45,46^ similar to the μM potency of xylazine inhibition of α7 nAChRs (Figure 1a). The impact of xylazine as a competitive antagonist of α7 nAChRs may have a similar weight to xylazine as an agonist of α2-ARs, though such a possibility requires thorough studies in the future. Second, α7 nAChRs are prominent players in both the central and peripheral nervous systems. They also play a major role in regulating the cholinergic anti-inflammatory pathway, a key mechanism by which the nervous system controls immune responses and inflammation.

Among all AChRs, α7 nAChRs are unique in their high Ca^2+^ permeability that is comparable to that of NMDA receptors.^14,60−62^ Activation of α7 nAChRs triggers an increase in [Ca^2+^]_in_ transients and subsequent calcium-dependent signaling through distinct cell populations.^12,14,63,64^ α7 nAChRs in neural cells influence cognition, memory, and behaviors relevant to symptoms observed in xylazine withdrawal, such as aggression, irritability, and anxiety.^65^ Serious deficits in cognitive functions and poor memory were detected in chronic xylazine users, even after detoxification.^66^ Xylazine also causes severe skin ulcers and abscesses, leading to infections, rotting tissue, and even amputations. The established link of xylazine to functional inhibition of α7 nAChRs by our studies is an important step in the treatment of clinically observed xylazine-associated symptoms.^12,14,63,65,66^ Previous preclinical studies and clinical trials showed promising benefits of α7 nAChR-selective agonists and PAMs in improving cognitive functions and memory, reducing aggression and anxiety, and countering various skin diseases.^12,67,68^ In addition to curcuminoids used in current studies, other α7 nAChR-selective agonists or PAMs are also potential candidates for counteracting xylazine and restoring the normal function of α7 nAChRs.

The curcuminoid potentiation of the function of α7 nAChRs is shown in both electrophysiology measurements of *Xenopus* oocytes expressing recombinant α7 nAChRs (Figures 2 and 3) and [Ca^2+^]_in_ fluorescence imaging of PC12 cells that endogenously express α7 nAChRs (Figure 5). These results are consistent with the reports that curcuminoids are effective positive allosteric modulators (PAMs) of α7 nAChRs.^44,69^ PAMs bind to a site in receptors that is different from the orthosteric site for binding agonists (e.g., acetylcholine and choline) and competitive antagonists (e.g., xylazine and α-bungarotoxin), enhance open-channel conformations stimulated by the agonists, and thereby amplify channel currents of α7 nAChRs.^70,71^ The recent structural and functional studies of α7 nAChRs in complex with PAMs^57,58^ illustrated a potential binding site for curcuminoids in the transmembrane domain of α7 nAChRs. Indeed, our docking studies (data are not shown) suggest that DMC can bind to α7 nAChRs at the site for ivermectin and the α7-selective PNU-120596.^57,58^ In principle, all the α7 nAChR PAMs can potentially reverse actions of antagonists (e.g., xylazine) on α7 nAChRs. However, none of the α7 nAChR-selective PAMs have progressed from clinical trials into therapeutic use yet, despite some of them showing promise in preclinical studies.^72^

It has been well demonstrated in the past decades that natural products are valuable sources of new drugs.^73^ Ivermectin, a nonselective PAM of α7 nAChRs, sets a good example. Isolated originally from woodland soil and evolved eventually into an FDA-approved drug, ivermectin is used in humans to treat river blindness and lymphatic filariasis as well as other health issues.^74,75^ Our new results demonstrate that ivermectin can effectively offset xylazine inhibitory effects on α7 nAChRs (Figures 2 and 3), providing a basis to repurpose ivermectin in countering xylazine-caused damage to human health. Similarly, as naturally occurring compounds in turmeric and effective PAMs of α7 nAChRs, curcuminoids are used by humans as dietary supplements and an adjunctive treatment for a broad spectrum of diseases and disorders.^76−79^ The neuroprotective and anti-inflammatory effects of curcuminoids against drugs of abuse are demonstrated.^80^ Their selectivity to α7 nAChRs over other subtypes of neuronal nAChRs was reported previously^44^ and exhibited in our studies (Figure S6). It is notable that 0.4–0.5 μM DMC was sufficient to reverse the xylazine (10 μM) inhibition of ACh-elicited currents from *Xenopus* oocytes expressing α7 nAChRs (Figure 3b) and [Ca^2+^]_in_ transients mediated by α7 nAChRs in PC12 cells (Figure 5). This finding is important for therapeutic applications, considering the low bioavailability of curcuminoids in their native form. The bioavailability of curcuminoids in both oral and topical administrations, however, has improved significantly due to recent advances in drug formulation and delivery.^50,79,81−83^ These progresses along with the proven safety of curcuminoids on humans^78,84^ open a path for future use of curcuminoids to reverse damages caused by xylazine-adulterated drugs.

The present studies established α7 nAChRs as molecular targets of xylazine, which competitively antagonizes the function of α7 nAChRs. The finding provides a basis to counteract xylazine actions with a focus on α7 nAChRs. The potency of xylazine on α7 nAChRs is comparable with that on α2-ARs^45,46^ and suggests that α7 nAChRs should be included along α2-ARs in the design of treatment strategies for xylazine-adulterated drug users. The possibility of xylazine targeting other subtypes of nAChRs, especially α4β2 nAChRs (Figures 6 and S5), has also been confirmed by our studies, though xylazine (10 μM) produces less profound inhibition on α4β2 nAChRs than on α7 nAChRs (Figures 3b and 6). In addition to identifying α7 nAChRs as xylazine targets, our studies also provide compelling evidence to support curcuminoids and ivermectin for reversing xylazine action mediated by α7 nAChRs but not by α4β2 nAChRs (Figures S6 and S7). As natural medicines, their medical benefits may result from interactions with a wide range of biochemical pathways and multiple molecular targets.^85^ Being PAMs of α7 nAChRs is probably one of their pharmacological actions. Future research should further exploit whether and how they, particularly curcuminoids, generate synergistic neuroprotective, anti-inflammatory, antioxidant, and antiapoptotic effects in combatting damages by xylazine-adulterated drugs.

## Materials and Methods

### Chemicals

All of the chemicals used in the experiments were purchased from Millipore Sigma if not stated otherwise. All of them were directly dissolved in recording solutions except for DMC, which was dissolved in DMSO before being diluted (1:1000) into recording solutions. Perfusing solutions used for live-cell imaging contain nifedipine (1 μM) to suppress the activities of voltage-gated calcium channels.

### Electrophysiology

The DNAs encoding the full-length human α7, α4, and β2 nAChRs were kindly provided by Lindstrom’s lab (University of Pennsylvania, PA). For expression in *Xenopus* oocytes, nAChRs DNAs were introduced into the oocyte expression vector pMXT(α7), pCMVT7(α4), and pBlueScript KS(β2) using the NEBuilder HiFi DNA Assembly Master Mix (NEB) and confirmed by DNA sequencing. The two-electrode voltage-clamp (TEVC) measurements of the channel function of α7 and α4β2 nAChRs in *Xenopus* oocytes were reported previously.^86−88^ Briefly, 25 ng of human α7 nAChR RNA was coinjected with 25 ng of RIC3 RNA into *Xenopus laevis* oocytes (stages 5–6). Channel functions of α7 nAChRs were measured ∼20 h after the RNA injection in a 20 μL oocyte recording chamber (Automate Scientific) clamped at −60 mV with an OC-725C Amplifier (Warner Instruments). In the case of α4β2 nAChRs, a total of 15 ng of RNA with an α4/β2 ratio of 1:2 or 2:1 was coinjected and channel functions were measured ∼48 h after the RNA injection. The perfusion rate of ∼2.4 mL/min was kept for ensuring complete buffer exchange every 0.5 s. The recording solutions contained 96 mM NaCl, 2 mM KCl, 1.8 mM CaCl_2_, 1 mM MgCl_2_, and 5 mM HEPES, pH 7.0, as well as indicated concentrations of the agonist acetylcholine, xylazine, and DMC. Clampex 10 software (Molecular Devices) was used for data collection and processing. Nonlinear regressions and statistical analysis were performed using GraphPad Prism 10.0 (GraphPad Software, Inc.).

### PC12 Cell Culture

PC12 cells (ATCC, CRL1721.1) were cultured in F12K media supplemented with 10% horse serum, 5% fetal bovine serum, and 1% penicillin/streptomycin/fungizone in an incubator with 5% CO_2_ and 80% relative humidity at 37 °C. The cells were maintained in a T75 flask, the medium was changed every 2–3 days, and the cells were passed at 60–80% confluency to the 0.1% poly-l-lysine-coated D35-10 glass-bottom dishes for calcium imaging.

### Calbryte 520 AM Loading in PC12 Cells

After overnight growth on a glass-bottom dish, PC12 cells were washed 3 times with 1 mL of modified Hanks’ balanced salt solution (HBSS, 5 mM KCl, 0.3 mM KH_2_PO_4_, 138 mM NaCl, 0.3 mM Na_2_HPO_4_, 4 mM NaHCO_3_, 5.6 mM glucose, 1 mM MgCl_2_, 2 mM CaCl_2_, and 10 mM HEPES, adjusted to pH 7.4 with 10 M NaOH). Stock solutions of Calbryte 520 AM (AAT Bioquest) and Pluronic F-127 were prepared in DMSO at 1 mM and 20% (w/v), respectively. The mixtures of 10 μL of Calbryte 520 AM, 8 μL of Pluronic F-127, and 4 mL of modified HBSS were prepared for 8 dishes of cells. Each dish was loaded with ∼500 μL of the mixture solution and incubated for ∼25 min in the dark at room temperature. The cells were washed three times with modified HBSS (1 mL) before calcium imaging.

### Live-Cell Fluorescence Calcium Imaging

The microscopic images were taken with an inverted Zeiss Observer Z1 microscope equipped with a motorized stage. All images were taken with a 20×/0.50 Zeiss Plan-Neofluar lens or 40×/0.90 Olympus lens (UPLSAPo40X). The perfusion system was controlled by a VC-8 valve controller (Warner Instruments). Reference images were collected from the Open and GFP channels before and after each perfusion experiment. The sequential images were recorded every two seconds at 528 nm after a GFP-3035B-OMF filter cube during each perfusion experiment. Image collection and perfusion synchronization were controlled by SlideBook 6 (Intelligent Imaging Innovations, Inc.).

### Data Extraction from Fluorescent Images and Analysis

The recorded images were exported in TIFF format by SlideBook Reader and further processed with ImageJ Fiji.^89^ The resulting GFP stack was aligned using StackReg with Rigid Body transformation.^90^ The region of interest (ROI) was identified based on reference images. The intensity of the individual ROI was extracted for the entire course of perfusion. ROIs with abnormal baseline changes, due to sudden cell death or debris floating across the field of view, were filtered in further data processing and analysis. Intensity changes of individual ROI were normalized to the intensity resulting from the first application of 10 mM choline, which served as a reference for each ROI. All data fitting and analysis were performed using GraphPad Prism 10.0, which was also used for statistical analysis. Statistical significance was determined by the extra sum of squares *F* test. A value of *p* < 0.05 is considered significant.

### Xylazine Docking to nAChRs

The structure of xylazine was obtained from PubChem (CID 5707). Xylazine docking was performed using AutoDock4.2.^91^ and nAChR structures of α7 (PDBID: 7KOX and 7EKT), α4β2 (PDBID: 8ST0 and 8ST2), and α3β4 (PDBID: 6PV7). The grid searching box was centered above the TMD/ICD interface of nAChRs with a box size of (160, 100, 120) at a spacing of 0.375 Å. A total of 500 docks were performed for each receptor structure, and free energies of binding were calculated using the Lamarckian genetic algorithm.

## Data Availability

Data will be made available on request.

## Supporting Information Available

Additional experimental results including α-bungarotoxin inhibition of α7nAChRs, representative fluorescence images of live PC12 cells loaded with Calbryte 520 AM under different perfused solutions, xylazine inhibition of increases in [Ca^2+^]_in_ transients elicited by acetylcholine, evaluation of autofluorescence resulting from DMC, xylazine docking results, DMC potentiating α7nAChRs but not α4β2nAChRs, and figure showing that ivermectin is not a PAM for α4β2nAChRs (PDF)

## References

[ref1] Gupta R.; Holtgrave D. R.; Ashburn M. A. Xylazine - Medical and Public Health Imperatives. N. Engl. J. Med. 2023, 388 (24), 2209–2212. 10.1056/NEJMp2303120. 37099338

[ref2] Holt A. C.; Schwope D. M.; Le K.; Schrecker J. P.; Heltsley R. Widespread Distribution of Xylazine Detected Throughout the United States in Healthcare Patient Samples. J. Addict. Med. 2023, 17 (4), 468–470. 10.1097/ADM.0000000000001132. 37579111 PMC10417214

[ref3] Malayala S. V.; Papudesi B. N.; Bobb R.; Wimbush A. Xylazine-Induced Skin Ulcers in a Person Who Injects Drugs in Philadelphia, Pennsylvania, USA. Cureus 2022, 14 (8), e28160 10.7759/cureus.28160. 36148197 PMC9482722

[ref4] Rubin R. Warning About Xylazine, a Veterinary Sedative Found in Illicit Drugs. JAMA 2022, 328 (23), 2296 10.1001/jama.2022.20045. 36538321

[ref5] Ehrman-Dupre R.; Kaigh C.; Salzman M.; Haroz R.; Peterson L. K.; Schmidt R. Management of Xylazine Withdrawal in a Hospitalized Patient: A Case Report. J. Addict. Med. 2022, 16 (5), 595–598. 10.1097/ADM.0000000000000955. 35020700

[ref6] Ayub S.; Parnia S.; Poddar K.; Bachu A. K.; Sullivan A.; Khan A. M.; Ahmed S.; Jain L. Xylazine in the Opioid Epidemic: A Systematic Review of Case Reports and Clinical Implications. Cureus 2023, 15 (3), e36864 10.7759/cureus.36864. 37009344 PMC10063250

[ref7] Schwartz D. D.; Clark T. P. Affinity of detomidine, medetomidine and xylazine for alpha-2 adrenergic receptor subtypes. J. Vet. Pharmacol. Ther. 1998, 21 (2), 107–111. 10.1046/j.1365-2885.1998.00113.x. 9597647

[ref8] Ruiz-Colon K.; Chavez-Arias C.; Diaz-Alcala J. E.; Martinez M. A. Xylazine intoxication in humans and its importance as an emerging adulterant in abused drugs: A comprehensive review of the literature. Forensic Sci. Int. 2014, 240, 1–8. 10.1016/j.forsciint.2014.03.015. 24769343

[ref9] Wang H.; Yu M.; Ochani M.; Amella C. A.; Tanovic M.; Susarla S.; Li J. H.; Wang H.; Yang H.; Ulloa L.; et al. Nicotinic acetylcholine receptor alpha7 subunit is an essential regulator of inflammation. Nature 2003, 421 (6921), 384–388. 10.1038/nature01339. 12508119

[ref10] de Jonge W. J.; Ulloa L. The alpha7 nicotinic acetylcholine receptor as a pharmacological target for inflammation. Br. J. Pharmacol. 2007, 151 (7), 915–929. 10.1038/sj.bjp.0707264. 17502850 PMC2042938

[ref11] O’Neil J.; Kovach S. Xylazine-Associated Skin Injury. N. Engl. J. Med. 2023, 388 (24), 2274 10.1056/NEJMicm2303601. 37314708

[ref12] Stegemann A.; Bohm M. Targeting the alpha7 nicotinic acetylcholine receptor-A novel road towards the future treatment of skin diseases. Exp. Dermatol. 2020, 29 (9), 924–931. 10.1111/exd.14173. 32780438

[ref13] Papke R. L.; Horenstein N. A. Therapeutic Targeting of alpha7 Nicotinic Acetylcholine Receptors. Pharmacol. Rev. 2021, 73 (3), 1118–1149. 10.1124/pharmrev.120.000097. 34301823 PMC8318519

[ref14] Letsinger A. C.; Gu Z.; Yakel J. L. alpha7 nicotinic acetylcholine receptors in the hippocampal circuit: taming complexity. Trends Neurosci. 2022, 45 (2), 145–157. 10.1016/j.tins.2021.11.006. 34916082 PMC8914277

[ref15] Bencherif M.; Lippiello P. M.; Lucas R.; Marrero M. B. Alpha7 nicotinic receptors as novel therapeutic targets for inflammation-based diseases. Cell. Mol. Life Sci. 2011, 68 (6), 931–949. 10.1007/s00018-010-0525-1. 20953658 PMC3678737

[ref16] Pavlov V. A.; Tracey K. J. Neural regulation of immunity: molecular mechanisms and clinical translation. Nat. Neurosci. 2017, 20 (2), 156–166. 10.1038/nn.4477. 28092663

[ref17] Schloss M. J.; Hulsmans M.; Rohde D.; Lee I. H.; Severe N.; Foy B. H.; Pulous F. E.; Zhang S.; Kokkaliaris K. D.; Frodermann V.; et al. B lymphocyte-derived acetylcholine limits steady-state and emergency hematopoiesis. Nat. Immunol. 2022, 23 (4), 605–618. 10.1038/s41590-022-01165-7. 35352063 PMC8989652

[ref18] Corradi J.; Bouzat C. Understanding the Bases of Function and Modulation of alpha7 Nicotinic Receptors: Implications for Drug Discovery. Mol. Pharmacol. 2016, 90 (3), 288–299. 10.1124/mol.116.104240. 27190210

[ref19] Fucile S.; Renzi M.; Lax P.; Eusebi F. Fractional Ca(2+) current through human neuronal alpha7 nicotinic acetylcholine receptors. Cell Calcium 2003, 34 (2), 205–209. 10.1016/S0143-4160(03)00071-X. 12810063

[ref20] Zanetti S. R.; Ziblat A.; Torres N. I.; Zwirner N. W.; Bouzat C. Expression and Functional Role of alpha7 Nicotinic Receptor in Human Cytokine-stimulated Natural Killer (NK) Cells. J. Biol. Chem. 2016, 291 (32), 16541–16552. 10.1074/jbc.M115.710574. 27284006 PMC4974370

[ref21] Dawe G. B.; Yu H.; Gu S.; Blackler A. N.; Matta J. A.; Siuda E. R.; Rex E. B.; Bredt D. S. alpha7 nicotinic acetylcholine receptor upregulation by anti-apoptotic Bcl-2 proteins. Nat. Commun. 2019, 10 (1), 2746 10.1038/s41467-019-10723-x. 31227712 PMC6588605

[ref22] Zdanowski R.; Krzyzowska M.; Ujazdowska D.; Lewicka A.; Lewicki S. Role of α7 nicotinic receptor in the immune system and intracellular signaling pathways. Cent. Eur. J. Immunol. 2015, 40 (3), 373–379. 10.5114/ceji.2015.54602. 26648784 PMC4655390

[ref23] Couturier S.; Bertrand D.; Matter J. M.; Hernandez M. C.; Bertrand S.; Millar N.; Valera S.; Barkas T.; Ballivet M. A neuronal nicotinic acetylcholine receptor subunit (alpha 7) is developmentally regulated and forms a homo-oligomeric channel blocked by alpha-BTX. Neuron 1990, 5 (6), 847–856. 10.1016/0896-6273(90)90344-F. 1702646

[ref24] Albuquerque E. X.; Pereira E. F.; Alkondon M.; Rogers S. W. Mammalian nicotinic acetylcholine receptors: from structure to function. Physiol. Rev. 2009, 89 (1), 73–120. 10.1152/physrev.00015.2008. 19126755 PMC2713585

[ref25] Jensen K. P.; DeVito E. E.; Yip S.; Carroll K. M.; Sofuoglu M. The Cholinergic System as a Treatment Target for Opioid Use Disorder. CNS Drugs 2018, 32 (11), 981–996. 10.1007/s40263-018-0572-y. 30259415 PMC6314885

[ref26] Lichenstein S. D.; Zakiniaeiz Y.; Yip S. W.; Garrison K. A. Mechanisms and Clinical Features of Co-occurring Opioid and Nicotine Use. Curr. Addict. Rep. 2019, 6 (2), 114–125. 10.1007/s40429-019-00245-3. 32864292 PMC7451029

[ref27] Atanasov A. G.; Zotchev S. B.; Dirsch V. M.; Orhan I. E.; Banach M.; Rollinger J. M.; Barreca D.; Weckwerth W.; Bauer R.; Bayer E. A.; et al. Natural products in drug discovery: advances and opportunities. Nat. Rev. Drug Discovery 2021, 20 (3), 200–216. 10.1038/s41573-020-00114-z. 33510482 PMC7841765

[ref28] Gupta S. C.; Patchva S.; Aggarwal B. B. Therapeutic roles of curcumin: lessons learned from clinical trials. AAPS J. 2013, 15 (1), 195–218. 10.1208/s12248-012-9432-8. 23143785 PMC3535097

[ref29] Basnet P.; Skalko-Basnet N. Curcumin: an anti-inflammatory molecule from a curry spice on the path to cancer treatment. Molecules 2011, 16 (6), 4567–4598. 10.3390/molecules16064567. 21642934 PMC6264403

[ref30] Lao C. D.; Ruffin M. T. t.; Normolle D.; Heath D. D.; Murray S. I.; Bailey J. M.; Boggs M. E.; Crowell J.; Rock C. L.; Brenner D. E. Dose escalation of a curcuminoid formulation. BMC Complementary Altern. Med. 2006, 6, 10 10.1186/1472-6882-6-10. PMC1434783 16545122

[ref31] Merecz-Sadowska A.; Sitarek P.; Sliwinski T.; Zajdel R. Anti-Inflammatory Activity of Extracts and Pure Compounds Derived from Plants via Modulation of Signaling Pathways, Especially PI3K/AKT in Macrophages. Int. J. Mol. Sci. 2020, 21 (24), 9605 10.3390/ijms21249605. 33339446 PMC7766727

[ref32] Shin S. A.; Joo B. J.; Lee J. S.; Ryu G.; Han M.; Kim W. Y.; Park H. H.; Lee J. H.; Lee C. S. Phytochemicals as Anti-Inflammatory Agents in Animal Models of Prevalent Inflammatory Diseases. Molecules 2020, 25 (24), 5932 10.3390/molecules25245932. 33333788 PMC7765227

[ref33] Ullah A.; Munir S.; Badshah S. L.; Khan N.; Ghani L.; Poulson B. G.; Emwas A. H.; Jaremko M. Important Flavonoids and Their Role as a Therapeutic Agent. Molecules 2020, 25 (22), 5243 10.3390/molecules25225243. 33187049 PMC7697716

[ref34] Oz M.; Lozon Y.; Sultan A.; Yang K. H.; Galadari S. Effects of monoterpenes on ion channels of excitable cells. Pharmacol. Ther. 2015, 152, 83–97. 10.1016/j.pharmthera.2015.05.006. 25956464

[ref35] Sahebkar A. Are curcuminoids effective C-reactive protein-lowering agents in clinical practice? Evidence from a meta-analysis. Phytother. Res. 2014, 28 (5), 633–642. 10.1002/ptr.5045. 23922235

[ref36] Aggarwal B. B.; Gupta S. C.; Sung B. Curcumin: an orally bioavailable blocker of TNF and other pro-inflammatory biomarkers. Br. J. Pharmacol. 2013, 169 (8), 1672–1692. 10.1111/bph.12131. 23425071 PMC3753829

[ref37] Yu J. J.; Pei L. B.; Zhang Y.; Wen Z. Y.; Yang J. L. Chronic Supplementation of Curcumin Enhances the Efficacy of Antidepressants in Major Depressive Disorder: A Randomized, Double-Blind, Placebo-Controlled Pilot Study. J. Clin. Psychopharmacol. 2015, 35 (4), 406–410. 10.1097/JCP.0000000000000352. 26066335

[ref38] Aggarwal B. B.; Harikumar K. B. Potential therapeutic effects of curcumin, the anti-inflammatory agent, against neurodegenerative, cardiovascular, pulmonary, metabolic, autoimmune and neoplastic diseases. Int. J. Biochem. Cell Biol. 2009, 41 (1), 40–59. 10.1016/j.biocel.2008.06.010. 18662800 PMC2637808

[ref39] Kuptniratsaikul V.; Dajpratham P.; Taechaarpornkul W.; Buntragulpoontawee M.; Lukkanapichonchut P.; Chootip C.; Saengsuwan J.; Tantayakom K.; Laongpech S. Efficacy and safety of Curcuma domestica extracts compared with ibuprofen in patients with knee osteoarthritis: a multicenter study. Clin. Interventions Aging 2014, 9, 451–458. 10.2147/CIA.S58535. PMC3964021 24672232

[ref40] Panahi Y.; Hosseini M. S.; Khalili N.; Naimi E.; Simental-Mendia L. E.; Majeed M.; Sahebkar A. Effects of curcumin on serum cytokine concentrations in subjects with metabolic syndrome: A post-hoc analysis of a randomized controlled trial. Biomed. Pharmacother. 2016, 82, 578–582. 10.1016/j.biopha.2016.05.037. 27470399

[ref41] Ramaholimihaso T.; Bouazzaoui F.; Kaladjian A. Curcumin in Depression: Potential Mechanisms of Action and Current Evidence-A Narrative Review. Front. Psychiatry 2020, 11, 572533 10.3389/fpsyt.2020.572533. 33329109 PMC7728608

[ref42] O’Sullivan S. J.; Malahias E.; Park J.; Srivastava A.; Reyes B. A. S.; Gorky J.; Vadigepalli R.; Van Bockstaele E. J.; Schwaber J. S. Single-Cell Glia and Neuron Gene Expression in the Central Amygdala in Opioid Withdrawal Suggests Inflammation With Correlated Gut Dysbiosis. Front. Neurosci. 2019, 13, 665 10.3389/fnins.2019.00665. 31333398 PMC6619439

[ref43] Lacagnina M. J.; Rivera P. D.; Bilbo S. D. Glial and Neuroimmune Mechanisms as Critical Modulators of Drug Use and Abuse. Neuropsychopharmacology 2017, 42 (1), 156–177. 10.1038/npp.2016.121. 27402494 PMC5143481

[ref44] El Nebrisi E. G.; Bagdas D.; Toma W.; Al Samri H.; Brodzik A.; Alkhlaif Y.; Yang K. S.; Howarth F. C.; Damaj I. M.; Oz M. Curcumin Acts as a Positive Allosteric Modulator of alpha(7)-Nicotinic Acetylcholine Receptors and Reverses Nociception in Mouse Models of Inflammatory Pain. J. Pharmacol. Exp. Ther. 2018, 365 (1), 190–200. 10.1124/jpet.117.245068. 29339457 PMC7947331

[ref45] Jasper J. R.; Lesnick J. D.; Chang L. K.; Yamanishi S. S.; Chang T. K.; Hsu S. A.; Daunt D. A.; Bonhaus D. W.; Eglen R. M. Ligand efficacy and potency at recombinant alpha2 adrenergic receptors: agonist-mediated [35S]GTPgammaS binding. Biochem. Pharmacol. 1998, 55 (7), 1035–1043. 10.1016/S0006-2952(97)00631-X. 9605427

[ref46] Biggane J. P.; Xu K.; Goldenstein B. L.; Davis K. L.; Luger E. J.; Davis B. A.; Jurgens C. W. D.; Perez D. M.; Porter J. E.; Doze V. A. Pharmacological characterization of the alpha(2A)-adrenergic receptor inhibiting rat hippocampal CA3 epileptiform activity: comparison of ligand efficacy and potency. J. Recept. Signal Transduction Res. 2022, 42 (6), 580–587. 10.1080/10799893.2022.2110896. PMC10710878 35984443

[ref47] de Jong L. A.; Uges D. R.; Franke J. P.; Bischoff R. Receptor-ligand binding assays: technologies and applications. J. Chromatogr. B Analyt. Technol. Biomed. Life Sci. 2005, 829 (1–2), 1–25. 10.1016/j.jchromb.2005.10.002. 16253574

[ref48] Krause R. M.; Buisson B.; Bertrand S.; Corringer P. J.; Galzi J. L.; Changeux J. P.; Bertrand D. Ivermectin: a positive allosteric effector of the alpha7 neuronal nicotinic acetylcholine receptor. Mol. Pharmacol. 1998, 53 (2), 283–294. 10.1124/mol.53.2.283. 9463487

[ref49] Hurst R. S.; Hajos M.; Raggenbass M.; Wall T. M.; Higdon N. R.; Lawson J. A.; Rutherford-Root K. L.; Berkenpas M. B.; Hoffmann W. E.; Piotrowski D. W.; et al. A novel positive allosteric modulator of the alpha7 neuronal nicotinic acetylcholine receptor: in vitro and in vivo characterization. J. Neurosci. 2005, 25 (17), 4396–4405. 10.1523/JNEUROSCI.5269-04.2005. 15858066 PMC6725110

[ref50] Hirose A.; Kuwabara Y.; Kanai Y.; Kato C.; Makino Y.; Yoshi F.; Sasaki K. Comparative pharmacokinetics of new curcumin preparations and evidence for increased bioavailability in healthy adult participants. Int. J. Clin. Pharmacol. Ther. 2022, 60 (12), 530–538. 10.5414/CP204257. 36278294 PMC9685553

[ref51] Musgrave I. F.; Krautwurst D.; Hescheler J.; Schultz G. Clonidine and cirazoline inhibit activation of nicotinic channels in PC-12 cells. Ann. N.Y. Acad. Sci. 1995, 763, 272–282. 10.1111/j.1749-6632.1995.tb32412.x. 7545885

[ref52] Janigro D.; Maccaferri G.; Meldolesi J. Calcium channels in undifferentiated PC12 rat pheochromocytoma cells. FEBS Lett. 1989, 255 (2), 398–400. 10.1016/0014-5793(89)81131-7. 2551740

[ref53] Chernyavsky A. I.; Arredondo J.; Skok M.; Grando S. A. Auto/paracrine control of inflammatory cytokines by acetylcholine in macrophage-like U937 cells through nicotinic receptors. Int. Immunopharmacol. 2010, 10 (3), 308–315. 10.1016/j.intimp.2009.12.001. 20004742 PMC2829366

[ref54] Kawashima K.; Fujii T.; Moriwaki Y.; Misawa H.; Horiguchi K. Non-neuronal cholinergic system in regulation of immune function with a focus on alpha7 nAChRs. Int. Immunopharmacol. 2015, 29 (1), 127–134. 10.1016/j.intimp.2015.04.015. 25907239

[ref55] Kim T. H.; Kim S. J.; Lee S. M. Stimulation of the alpha7 nicotinic acetylcholine receptor protects against sepsis by inhibiting Toll-like receptor via phosphoinositide 3-kinase activation. J. Infect. Dis. 2014, 209 (10), 1668–1677. 10.1093/infdis/jit669. 24298024

[ref56] Walsh R. M. Jr.; Roh S. H.; Gharpure A.; Morales-Perez C. L.; Teng J.; Hibbs R. E. Structural principles of distinct assemblies of the human alpha4beta2 nicotinic receptor. Nature 2018, 557 (7704), 261–265. 10.1038/s41586-018-0081-7. 29720657 PMC6132059

[ref57] Bondarenko V.; Chen Q.; Singewald K.; Haloi N.; Tillman T. S.; Howard R. J.; Lindahl E.; Xu Y.; Tang P. Structural Elucidation of Ivermectin Binding to alpha7nAChR and the Induced Channel Desensitization. ACS Chem. Neurosci. 2023, 14 (6), 1156–1165. 10.1021/acschemneuro.2c00783. 36821490 PMC10020961

[ref58] Zhao Y.; Liu S.; Zhou Y.; Zhang M.; Chen H.; Eric Xu H.; Sun D.; Liu L.; Tian C. Structural basis of human alpha7 nicotinic acetylcholine receptor activation. Cell Res. 2021, 31 (6), 713–716. 10.1038/s41422-021-00509-6. 33958730 PMC8169775

[ref59] D’Orazio J.; Nelson L.; Perrone J.; Wightman R.; Haroz R. Xylazine Adulteration of the Heroin-Fentanyl Drug Supply: A Narrative Review. Ann. Intern. Med. 2023, 176, 1370 10.7326/M23-2001. 37812779

[ref60] Seguela P.; Wadiche J.; Dineley-Miller K.; Dani J. A.; Patrick J. W. Molecular cloning, functional properties, and distribution of rat brain alpha 7: a nicotinic cation channel highly permeable to calcium. J. Neurosci. 1993, 13 (2), 596–604. 10.1523/JNEUROSCI.13-02-00596.1993. 7678857 PMC6576637

[ref61] Uteshev V. V. alpha7 nicotinic ACh receptors as a ligand-gated source of Ca(2+) ions: the search for a Ca(2+) optimum. Adv. Exp. Med. Biol. 2012, 740, 603–638. 10.1007/978-94-007-2888-2_27. 22453962 PMC3584641

[ref62] Shen J. X.; Yakel J. L. Nicotinic acetylcholine receptor-mediated calcium signaling in the nervous system. Acta Pharmacol. Sin. 2009, 30 (6), 673–680. 10.1038/aps.2009.64. 19448647 PMC4002362

[ref63] Kalkman H. O.; Feuerbach D. Modulatory effects of alpha7 nAChRs on the immune system and its relevance for CNS disorders. Cell. Mol. Life Sci. 2016, 73 (13), 2511–2530. 10.1007/s00018-016-2175-4. 26979166 PMC4894934

[ref64] Cheng Q.; Yakel J. L. The effect of alpha7 nicotinic receptor activation on glutamatergic transmission in the hippocampus. Biochem. Pharmacol. 2015, 97 (4), 439–444. 10.1016/j.bcp.2015.07.015. 26212541 PMC4600449

[ref65] Lewis A. S.; Picciotto M. R. Regulation of aggressive behaviors by nicotinic acetylcholine receptors: Animal models, human genetics, and clinical studies. Neuropharmacology 2020, 167, 107929 10.1016/j.neuropharm.2019.107929. 32058178 PMC7080580

[ref66] Mulders P.; van Duijnhoven V.; Schellekens A. Xylazine Dependence and Detoxification: A Case Report. Psychosomatics 2016, 57 (5), 529–533. 10.1016/j.psym.2016.05.001. 27480943

[ref67] Lewis A. S.; van Schalkwyk G. I.; Bloch M. H. Alpha-7 nicotinic agonists for cognitive deficits in neuropsychiatric disorders: A translational meta-analysis of rodent and human studies. Prog. Neuropsychopharmacol. Biol. Psychiatry 2017, 75, 45–53. 10.1016/j.pnpbp.2017.01.001. 28065843 PMC5446073

[ref68] Lewis A. S.; Olincy A.; Buchanan R. W.; Kem W. R.; Picciotto M. R.; Freedman R. Effects of a nicotinic agonist on the Brief Psychiatric Rating Scale five-factor subscale model in schizophrenia. Schizophr. Res. 2018, 195, 568–569. 10.1016/j.schres.2017.10.016. 29050790 PMC6476180

[ref69] Ximenis M.; Mulet J.; Sala S.; Sala F.; Criado M.; Gonzalez-Muniz R.; Perez de Vega M. J. Natural Polyhydroxy Flavonoids, Curcuminoids, and Synthetic Curcumin Analogs as alpha7 nAChRs Positive Allosteric Modulators. Int. J. Mol. Sci. 2021, 22 (2), 973 10.3390/ijms22020973. 33478095 PMC7835927

[ref70] Gulsevin A.; Papke R. L.; Stokes C.; Garai S.; Thakur G. A.; Quadri M.; Horenstein N. A. Allosteric Agonism of alpha7 Nicotinic Acetylcholine Receptors: Receptor Modulation Outside the Orthosteric Site. Mol. Pharmacol. 2019, 95 (6), 606–614. 10.1124/mol.119.115758. 30944209 PMC6491904

[ref71] Uteshev V. V. Allosteric Modulation of Nicotinic Acetylcholine Receptors: The Concept and Therapeutic Trends. Curr. Pharm. Des. 2016, 22 (14), 1986–1997. 10.2174/1381612822666160201115341. 26831463

[ref72] Sanders V. R.; Millar N. S. Potentiation and allosteric agonist activation of alpha7 nicotinic acetylcholine receptors: binding sites and hypotheses. Pharmacol. Res. 2023, 191, 106759 10.1016/j.phrs.2023.106759. 37023990

[ref73] Newman D. J.; Cragg G. M. Natural Products as Sources of New Drugs over the Nearly Four Decades from 01/1981 to 09/2019. J. Nat. Prod. 2020, 83 (3), 770–803. 10.1021/acs.jnatprod.9b01285. 32162523

[ref74] Molyneux D. H.; Ward S. A. Reflections on the Nobel Prize for Medicine 2015--The Public Health Legacy and Impact of Avermectin and Artemisinin. Trends Parasitol. 2015, 31 (12), 605–607. 10.1016/j.pt.2015.10.008. 26552892

[ref75] Laing R.; Gillan V.; Devaney E. Ivermectin - Old Drug, New Tricks?. Trends Parasitol. 2017, 33 (6), 463–472. 10.1016/j.pt.2017.02.004. 28285851 PMC5446326

[ref76] Eke-Okoro U. J.; Raffa R. B.; Pergolizzi J. V. Jr.; Breve F.; Taylor R. Jr.; NEMA Research Group Curcumin in turmeric: Basic and clinical evidence for a potential role in analgesia. J. Clin. Pharm. Ther. 2018, 43 (4), 460–466. 10.1111/jcpt.12703. 29722036

[ref77] Goozee K. G.; Shah T. M.; Sohrabi H. R.; Rainey-Smith S. R.; Brown B.; Verdile G.; Martins R. N. Examining the potential clinical value of curcumin in the prevention and diagnosis of Alzheimer’s disease. Br. J. Nutr. 2016, 115 (3), 449–465. 10.1017/S0007114515004687. 26652155

[ref78] Weng W.; Goel A. Curcumin and colorectal cancer: An update and current perspective on this natural medicine. Semin. Cancer Biol. 2022, 80, 73–86. 10.1016/j.semcancer.2020.02.011. 32088363 PMC7438305

[ref79] Vollono L.; Falconi M.; Gaziano R.; Iacovelli F.; Dika E.; Terracciano C.; Bianchi L.; Campione E. Potential of Curcumin in Skin Disorders. Nutrients 2019, 11 (9), 2169 10.3390/nu11092169. 31509968 PMC6770633

[ref80] Soltaninejad M.; Amleshi R. S.; Shabani M.; Ilaghi M. Unraveling the protective effects of curcumin against drugs of abuse. Heliyon 2024, 10 (9), e30468 10.1016/j.heliyon.2024.e30468. 38726155 PMC11079105

[ref81] Chelimela N.; Alavala R. R.; Satla S. R. Curcumin - Bioavailability Enhancement by Prodrug Approach and Novel Formulations. Chem. Biodivers. 2024, 21 (5), e202302030 10.1002/cbdv.202302030. 38401117

[ref82] Matthewman C.; Krishnakumar I. M.; Swick A. G. Review: bioavailability and efficacy of ’free’ curcuminoids from curcumagalactomannoside (CGM) curcumin formulation. Nutr. Res. Rev. 2024, 37 (1), 14–31. 10.1017/S0954422423000033. 36655498

[ref83] Kasprzak-Drozd K.; Nizinski P.; Hawryl A.; Gancarz M.; Hawryl D.; Oliwa W.; Palka M.; Markowska J.; Oniszczuk A. Potential of Curcumin in the Management of Skin Diseases. Int. J. Mol. Sci. 2024, 25 (7), 3617 10.3390/ijms25073617. 38612433 PMC11012053

[ref84] Howells L. M.; Iwuji C. O. O.; Irving G. R. B.; Barber S.; Walter H.; Sidat Z.; Griffin-Teall N.; Singh R.; Foreman N.; Patel S. R.; et al. Curcumin Combined with FOLFOX Chemotherapy Is Safe and Tolerable in Patients with Metastatic Colorectal Cancer in a Randomized Phase IIa Trial. J. Nutr. 2019, 149 (7), 1133–1139. 10.1093/jn/nxz029. 31132111 PMC6602900

[ref85] Akaberi M.; Sahebkar A.; Emami S. A. Turmeric and Curcumin: From Traditional to Modern Medicine. Adv. Exp. Med. Biol. 2021, 1291, 15–39. 10.1007/978-3-030-56153-6_2. 34331682

[ref86] Tillman T. S.; Choi Z.; Xu Y.; Tang P. Functional Tolerance to Cysteine Mutations in Human alpha7 Nicotinic Acetylcholine Receptors. ACS Chem. Neurosci. 2020, 11 (3), 242–247. 10.1021/acschemneuro.9b00647. 31951367 PMC7057392

[ref87] Tillman T. S.; Seyoum E.; Mowrey D. D.; Xu Y.; Tang P. ELIC-alpha7 Nicotinic acetylcholine receptor (alpha7nAChR) chimeras reveal a prominent role of the extracellular-transmembrane domain interface in allosteric modulation. J. Biol. Chem. 2014, 289 (20), 13851–13857. 10.1074/jbc.M113.524611. 24695730 PMC4022858

[ref88] Tillman T. S.; Alvarez F. J.; Reinert N. J.; Liu C.; Wang D.; Xu Y.; Xiao K.; Zhang P.; Tang P. Functional Human alpha7 Nicotinic Acetylcholine Receptor (nAChR) Generated from Escherichia coli. J. Biol. Chem. 2016, 291 (35), 18276–18282. 10.1074/jbc.M116.729970. 27385587 PMC5000075

[ref89] Schindelin J.; Arganda-Carreras I.; Frise E.; Kaynig V.; Longair M.; Pietzsch T.; Preibisch S.; Rueden C.; Saalfeld S.; Schmid B.; et al. Fiji: an open-source platform for biological-image analysis. Nat. Methods 2012, 9 (7), 676–682. 10.1038/nmeth.2019. 22743772 PMC3855844

[ref90] Thevenaz P.; Ruttimann U. E.; Unser M. A pyramid approach to subpixel registration based on intensity. IEEE Trans. Image Process 1998, 7 (1), 27–41. 10.1109/83.650848. 18267377

[ref91] Morris G. M.; Huey R.; Lindstrom W.; Sanner M. F.; Belew R. K.; Goodsell D. S.; Olson A. J. AutoDock4 and AutoDockTools4: Automated docking with selective receptor flexibility. J. Comput. Chem. 2009, 30 (16), 2785–2791. 10.1002/jcc.21256. 19399780 PMC2760638

