# Supplementary material for: Competitive Antagonism of Xylazine on α7 Nicotinic Acetylcholine Receptors and Reversal by Curcuminoids
Source: ACS Chem Neurosci. 2024 Dec 25;16(2):232–40. doi: 10.1021/acschemneuro.4c00784 (PMC11741004; doi:10.1021/acschemneuro.4c00784)
Supplement: Supplementary file 1 — cn4c00784_si_001.pdf [file cn4c00784_si_001.pdf]

Supporting Information

Competitive Antagonism of Xylazine on α7 Nicotinic Acetylcholine Receptors and Reversal by Curcuminoids

Qiang Chen[1], Yan Xu[1,2,3,4], Pei Tang[1,3*]

[1]Depatment of Anesthesiology and Perioperative Medicine, University of Pittsburgh, Pittsburgh, PA 15260, USA
[2]Department of Structural Biology, University of Pittsburgh, Pittsburgh, PA 15260, USA
[3]Department of Pharmacology and Chemical Biology, University of Pittsburgh, Pittsburgh, PA 15260, USA
[4]Department of Physics and Astronomy, University of Pittsburgh, Pittsburgh, PA 15260, USA

[*]Correspondence and requests for materials should be addressed to P.T. (email: ptang@pitt.edu)

There are seven supporting figures.

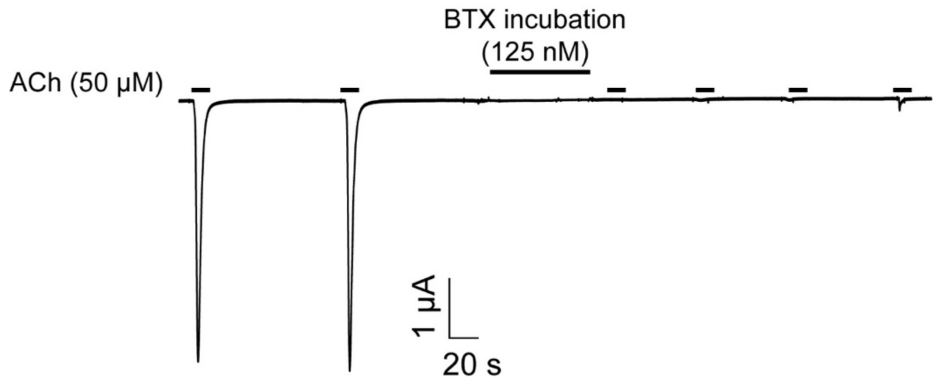

Figures S1. *α*-Bungarotoxin (BTX, 125 nM, an α7nAChR-selective antagonist) completely inhibited the ACh-induced current from *Xenopus* oocytes expressing α7nAChR, confirming that the observed current results from α7nAChR activation.

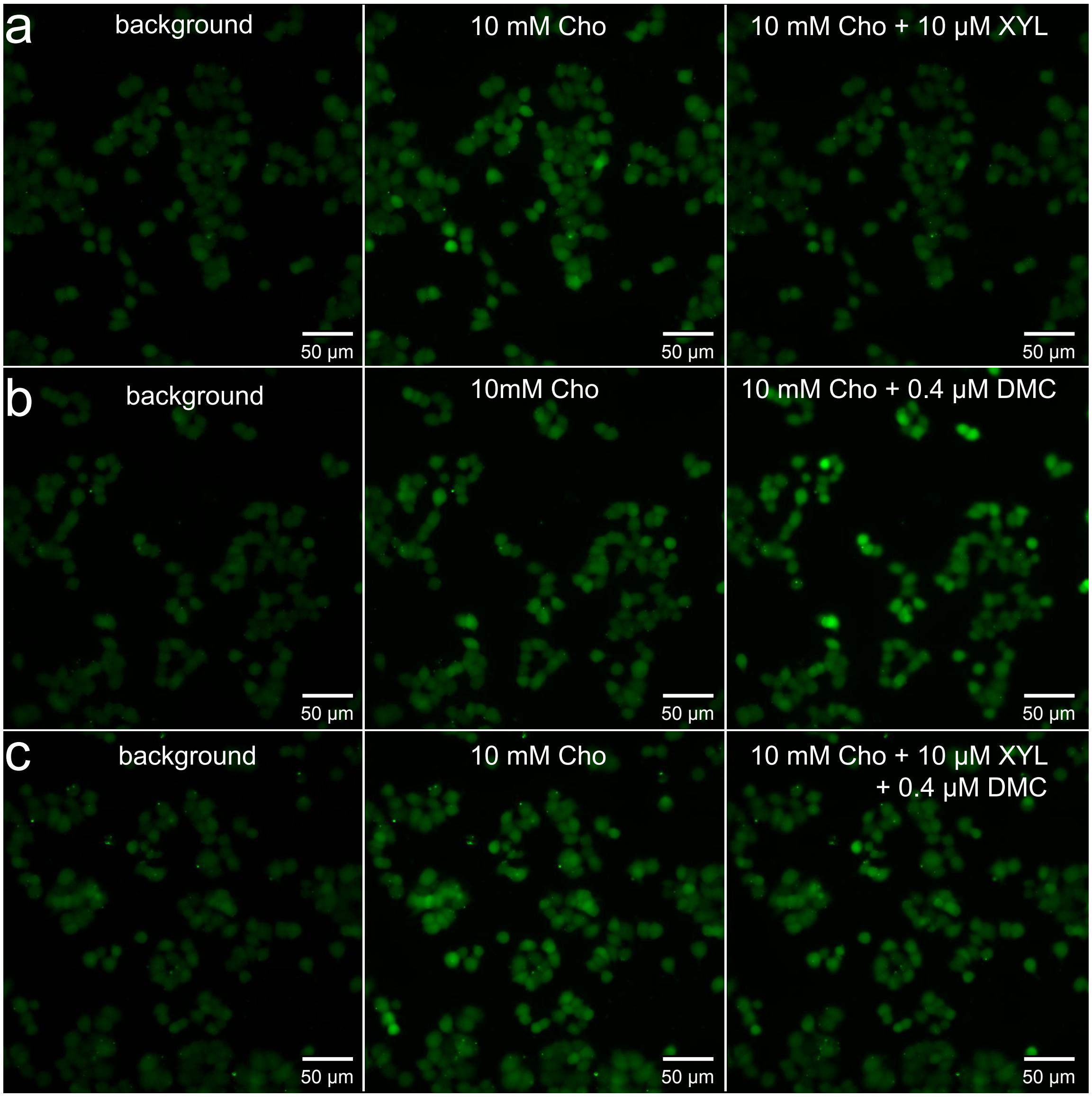

Figures S2. Representative images of live PC12 cells load with Calbryte™ 520 AM under different perfused solutions. (**a**) Choline (Cho) activation of α7nAChR and xylazine (XYL) inhibition as shown in changes of cell fluorescence: (*left*) Basal fluorescence, (*middle*) after applying 10 mM Cho, (*right*) after applying 10 mM Cho + 10 µM XYL. (**b**) DMC potentiation of Cho-stimulated $[Ca^{2+}]_{in}$ transient: (*left*) Basal fluorescence, (*middle*) after applying 10 mM Cho, (*right*) after applying 10 mM Cho + 0.4 µM DMC. (**c**) DMC recovers XYL-induced decrease of $[Ca^{2+}]_{in}$ transient: (*left*) Basal fluorescence, (*middle*) after applying 10 mM Cho, (*right*) after applying 10 mM Cho + 10 µM XYL + 0.4 µM DMC. Note that images in each row were obtained from the same population of cells.

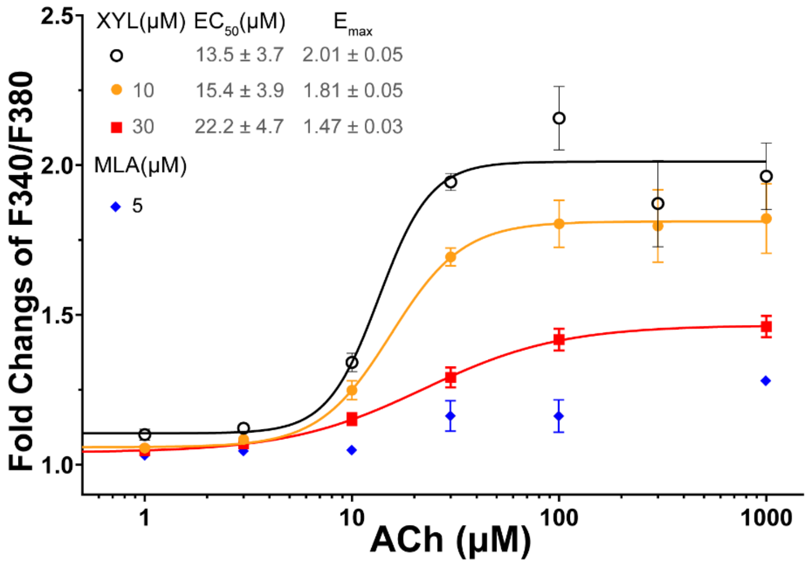

Figures S3. Xylazine inhibits $[Ca^{2+}]_{in}$ increases elicited by acetylcholine (ACh) activation of endogenous α7nAChRs in PC12 cells loaded with Fura-2AM in a dose-dependent manner. Increases in $[Ca^{2+}]_{in}$ transients are plotted as a function of ACh concentrations, measured by the F340/F380 ratio of Fura-2 AM (MilliporeSigma) loaded in PC12 cells. The presence of xylazine (10 μM or 30 μM) significantly inhibits the ACh-elicited $[Ca^{2+}]_{in}$ transients. The α7nAChR-selective antagonist MLA (5μM) largely, but not completely, inhibits the $[Ca^{2+}]_{in}$ transients, reflecting that unlike choline, ACh is a non-specific agonist to α7nAChRs and ACh-elicited $[Ca^{2+}]_{in}$ transients also result from receptors other than α7nAChR. The data fitting to the Hill equation resulted in EC_{50} and E_{max} (maximum efficacy). Data points are presented as mean ± SEM ($n \geq 70$ cells). Two-way ANOVA mixed-effects analysis shows significant xylazine effects (10 μM, $p=0.01$; 30 μM, $p<0.0001$).

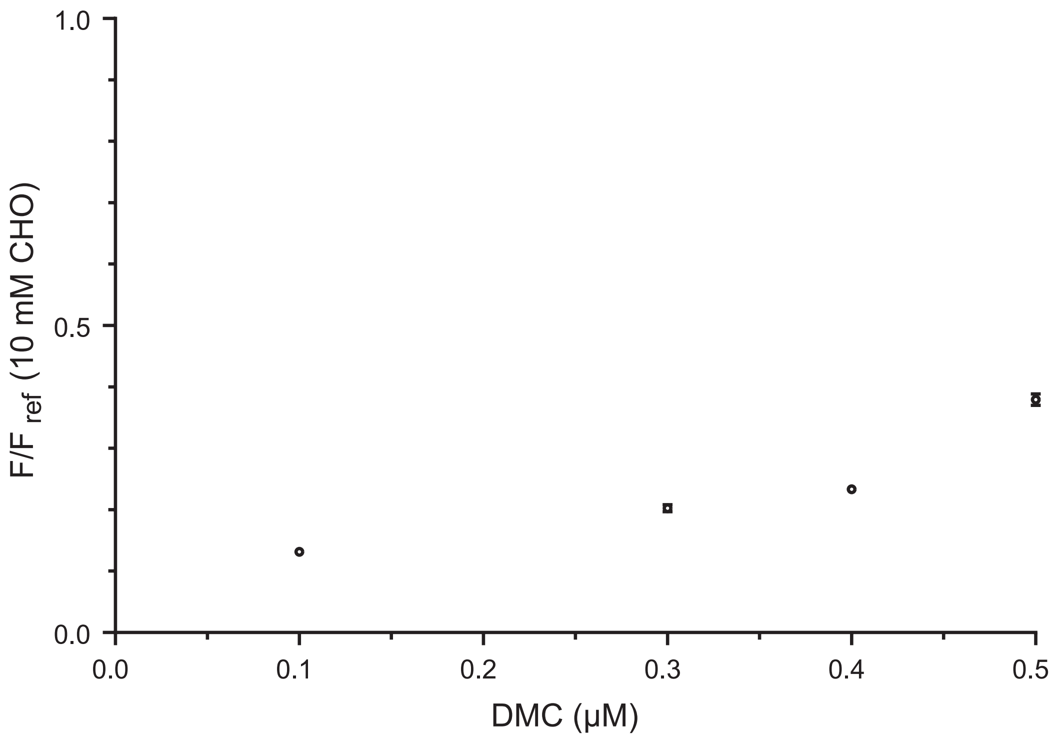

Figures S4. Evaluation of autofluorescence resulting from DMC. DMC-produced autofluorescence in the absence of receptor agonists is measured by the normalized fluorescence (F/F_{ref}) as a function of DMC concentrations, where F is the autofluorescence at a given DMC concentration in the absence of Cho; and F_{ref} is the fluorescence at 10 mM Cho without DMC. Data are presented as mean ± SEM (n = 249 cells from 8 different dishes). Note that 0.4 µM DMC was selected for our experiments involving DMC because this concentration showed minor autofluorescence (0.23) but effective potentiation of α7nAChR while counteracting xylazine's inhibitory effects (Fig. 5).

a

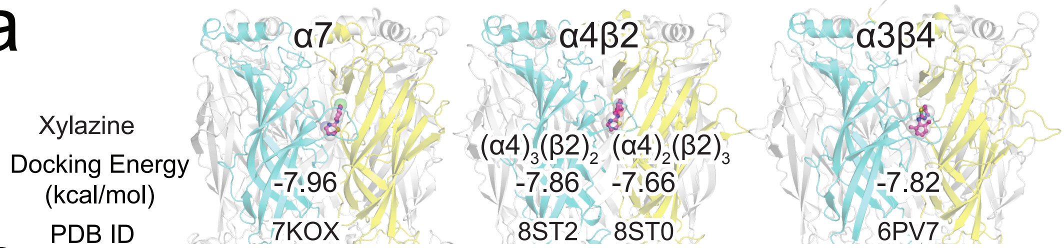

b

```
                                                                     55
α7    2   ----EFQRKLYKELVKN--YNPLERPVANDSQPLTVYFSLSLLQIMDVDEKNQVLTTNIWLQMSWTDHYL   65
α4    3   ETRAHAEERLLKKLFSG--YNKWSRPVANISDVVLVRFGLSIAQLIDVDEKNQMMTTNVWVKQEWHDYKL   70
β2    1   ---TDTEERLVEHLLDPSRYNKLIRPATNGSELVTVQLMVSLAQLISVHEREQIMTTNVWLTQEWEDYRL   67
α3    1   ---SEAEHRLFERLFED--YNEIIRPVANVSDPVIIHFEVSMSQLVKVDEVNQIMETNLWLKQIWNDYKL   65
β4    1   -RVANAEEKLMDDLLNKTRYNNLIRPATSSSQLISIKLQLSLAQLISVNEREQIMTTNVWLKQEWTDYRL   69

                                     93             106              119
α7   66   QWNVSEYPGVKTVRFPDGQIWKPDILLYNSADERFDATFHTNVLVNSSGHCQYLPPGIFKSSCYIDVRWF  135
α4   71   RWDPADYENVTSIRIPSELIWRPDIVLYNNADGDFAVTHLTKAHLFHDGRVQWTPPAIYKSSCSIDVTFF  140
β2   68   TWKPEEFDNMKKVRLPSKHIWLPDVVLYNNADGMYEVSFYSNAVVSYDGSIFWLPPAIYKSACKIEVKHF  137
α3   66   KWNPSDYGGAEFMRVPAQKIWKPDIVLYNNAVGDFQVDDKTKALLKYTGEVTWIPPAIFKSSCKIDVTYF  135
β4   70   TWNSSRYEGVNILRIPAKRIWLPDIVLYNNADGTYEVSVYTNLIVRSNGSVLWLPPAIYKSACKIEVKYF  139

                       149                                          191 195
α7  136   PFDVQHCKLKFGSWSYGGWSLDLQMQEA--DISGYIPNGEWDLVGIPGKRSERFYECCKEPYPDVTFTVT  203
α4  141   PFDQQNCTMKFGSWTYDKAKIDLVNMHSRVDQLDFWESGEWVIVDAVGTYNTRKYECCAEIYPDITYAFV  210
β2  138   PFDQQNCTMKFRSWTYDRTEIDLVLKSEVASLDDFTPSGEWDIVALPGRRNEN---PDDSTYVDITYDFI  204
α3  136   PFDYQNCTMKFGSWSYDKAKIDLVLIGSSMNLKDYWESGEWAIIKAPGYKHDIKYNCCEEIYPDITYSLY  205
β4  140   PFDQQNCTLKFRSWTYDHTEIDMVLMTPTASMDDFTPSGEWDIVALPGRRTVN---PQDPSYVDVTYDFI  206
```

Figures S5. Xylazine docking results on α7, α4β2, and α3β4 nAChRs and extracellular domain sequence alignment of these receptors. (a) AutoDock[1] reveals that xylazine (molecule in magenta) binds to the orthosteric ligand site in the extracellular domain (ECD) of these receptors. The average docking energies are marked. (**b**) The sequence alignment of the ECDs covering the orthosteric ligand site. The α7 residues within 3.5 Å of xylazine (marked in red) and aligned with corresponding residues in α4, β2, α3, and β4. The non-identical residues embracing the xylazine binding site (marked in green) likely contribute to the varied docking energies among different subtypes of nAChRs. A lower docking energy of xylazine in α7nAChR than α4β2nAChRs is consistent with the experimental finding that xylazine introduces a greater change of EC_{50} in the agonist ACh in α7nAChR. In addition, the docking results and sequence alignment suggest that xylazine is also likely to inhibit α3β4 nAChRs as it does on α7nAChR and α4β2nAChRs.

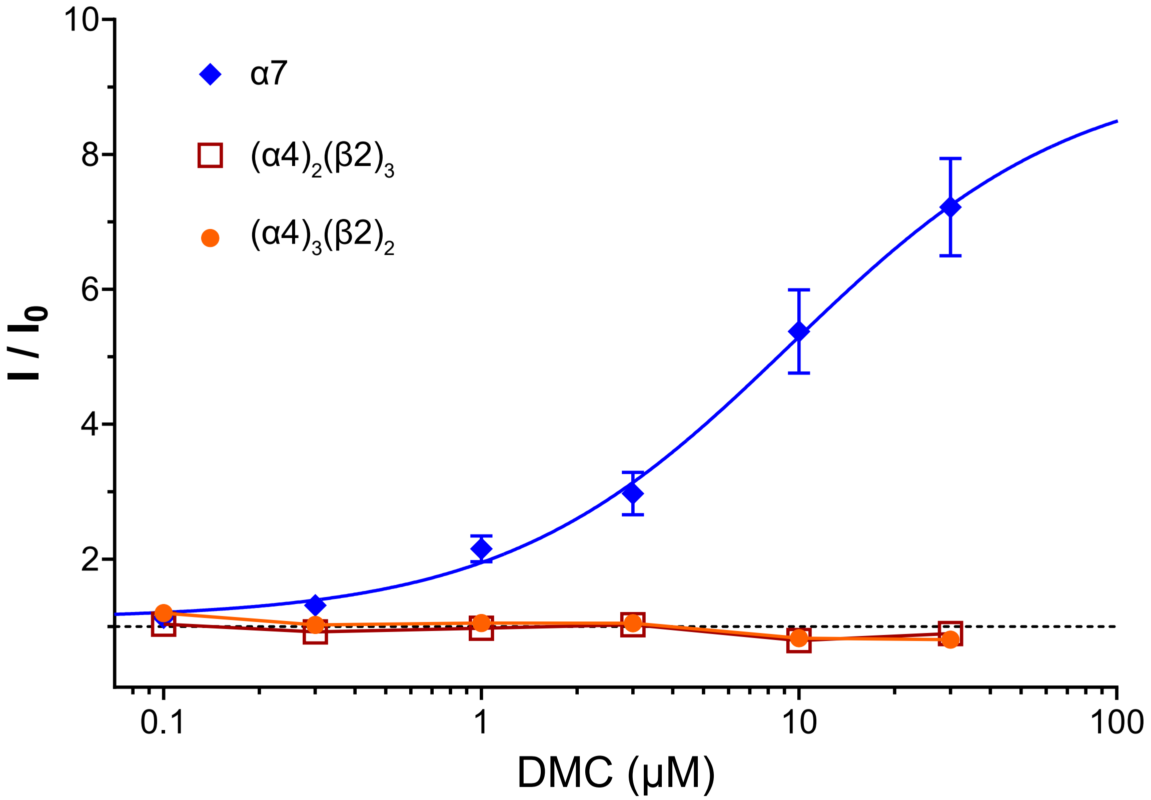

Figures S6. DMC potentiates α7nAChRs but not α4β2nAChRs. DMC, preincubated for 30 s before co-application with acetylcholine (EC_{10}, 30 μM for α7 and 1 μM for α4β2), potentiates currents of α7nAChRs in a dose-dependent manner but does not show potentiation effects on $(\alpha4)_2(\beta2)_3$ and $(\alpha4)_3(\beta2)_2$. All data were normalized to the current elicited by acetylcholine in the absence of DMC (I_0) and presented as mean ± SEM. Each data set was generated from $n \geq 7$ oocytes from two different donors.

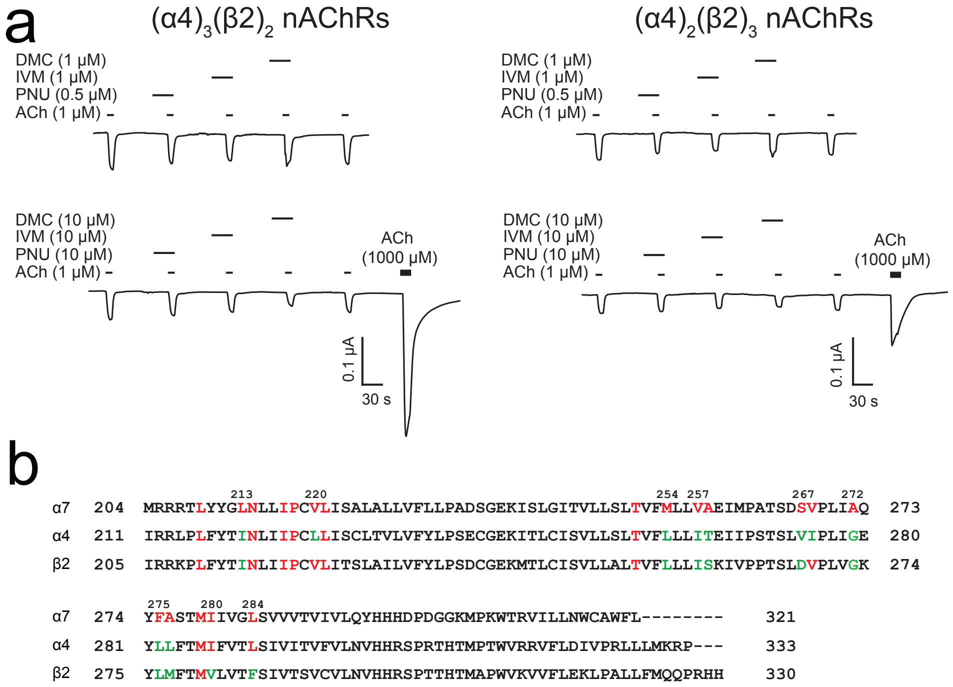

Figures S7. Ivermectin is not a PAM for α4β2nAChRs. (**a**) Representative current traces of $(\alpha4)_3(\beta2)_2$ and $(\alpha4)_2(\beta2)_3$ elicited by acetylcholine (ACh, 1 μM) in the absence and presence of ivermectin (IVM, 1 or 10 μM) or DMC (1 or 10 μM) or PNU120596 (PNU, 0.5 or 10 μM) that is a known α7-specific PAM but not a PAM of α4β2nAChRs.[2] As expected, PNU120596 does not produce potentiation of $(\alpha4)_3(\beta2)_2$ and $(\alpha4)_2(\beta2)_3$ currents. Consistent with the results shown in **Figures S6**, DMC shows no potentiation of $(\alpha4)_3(\beta2)_2$ and $(\alpha4)_2(\beta2)_3$. Like PNU120596 and DMC, ivermectin does not show positive modulation of $(\alpha4)_3(\beta2)_2$ and $(\alpha4)_2(\beta2)_3$, either. (**b**) The sequence alignment of the transmembrane domain embedding the ivermectin binding site in α7nAChRs shows differences between key α7 residues binding residues and those at equivalent positions of α4 or β2. These differences may impede ivermectin binding to $(\alpha4)_3(\beta2)_2$ and $(\alpha4)_2(\beta2)_3$. The results shown in (a) were confirmed by different *Xenopus* oocytes expressing $(\alpha4)_3(\beta2)_2$ (n = 5) and $(\alpha4)_2(\beta2)_3$ (n = 3).

References

1. Morris, G. M., Huey, R., Lindstrom, W., Sanner, M. F., Belew, R. K., Goodsell, D. S., and Olson, A. J. (2009) AutoDock4 and AutoDockTools4: Automated docking with selective receptor flexibility, *J Comput Chem 30*, 2785-2791.

2. Hurst, R. S., Hajos, M., Raggenbass, M., Wall, T. M., Higdon, N. R., Lawson, J. A., Rutherford-Root, K. L., Berkenpas, M. B., Hoffmann, W. E., Piotrowski, D. W., Groppi, V. E., Allaman, G., Ogier, R., Bertrand, S., Bertrand, D., and Arneric, S. P. (2005) A novel positive allosteric modulator of the alpha7 neuronal nicotinic acetylcholine receptor: in vitro and in vivo characterization, *J Neurosci 25*, 4396-4405.
